# Short-term metreleptin treatment of patients with anorexia nervosa: rapid on-set of beneficial cognitive, emotional, and behavioral effects

**DOI:** 10.1038/s41398-020-00977-1

**Published:** 2020-08-27

**Authors:** Gabriella Milos, Jochen Antel, Lisa-Katrin Kaufmann, Nikolaus Barth, Antonia Koller, Susanne Tan, Urban Wiesing, Anke Hinney, Lars Libuda, Martin Wabitsch, Roland von Känel, Johannes Hebebrand

**Affiliations:** 1 Eating Disorders Unit, Department of Consultation-Liaison Psychiatry and Psychosomatic Medicine, University Hospital of Zurich, University of Zurich, Zurich, Switzerland; 2 Department of Child and Adolescent Psychiatry, Psychosomatics, and Psychotherapy, University Hospital Essen (LVR-Klinikum), University of Duisburg-Essen, Essen, Germany; 3 Department of Endocrinology, Diabetes and Metabolism, University Hospital Essen, University Duisburg-Essen, Essen, Germany; 4 Institute for Ethics and History of Medicine, University of Tübingen, Tübingen, Germany; 5 Center for Rare Endocrine Diseases, Division of Pediatric Endocrinology and Diabetes, Department of Pediatrics and Adolescent Medicine, Ulm University Medical Center, Ulm, Germany

**Keywords:** Psychiatric disorders, Scientific community

## Abstract

To examine the hypothesis that normalization of low circulating leptin levels in patients with anorexia nervosa ameliorates hyperactivity, three seriously ill females with hyperactivity were treated off-label with metreleptin (recombinant human leptin) for up to 14 days. Drive for activity, repetitive thoughts of food, inner restlessness, and weight phobia decreased in two patients. Surprisingly, depression improved rapidly in all patients. No serious adverse events occurred. Due to obvious limitations of uncontrolled case series, placebo-controlled clinical trials are mandatory to confirm the observed rapid onset of beneficial effects. Our findings suggest an important role of hypoleptinemia in the mental and behavioral phenotype of anorexia nervosa.

## Introduction

Underweight, fear of weight gain, and body image disturbances represent cardinal features of anorexia nervosa (AN)^1,2^. Somatic, mental and behavioral symptoms of this eating disorder are intertwined with those of starvation^3^, which develops from the prolonged energy restriction characteristic of AN^2,4^. Loss of fat mass in AN entails a drop in blood levels of the adipocyte-derived hormone leptin^5^. Ensuing hypoleptinemia represents a key endocrine feature of this eating disorder^3,6,7^ and acts as the major signal for the adaptation to starvation^7–10^. Amenorrhea, hematological alterations, depressed mood, inflexibility, and repetitive thoughts of food represent clinically relevant examples of starvation-related symptoms, which might be triggered or worsened by hypoleptinemia^5^.

Hyperactivity is a common behavioral manifestation of AN, which has also been linked to hypoleptinemia^11–14^, albeit not consistently so^15^. Rodent studies point to a causal link between hyperactivity and low circulating leptin levels^11,16–18^. In rats, semi-starvation induced hyperactivity proved to be rapidly amenable to treatment with recombinant leptin in one study^11^, but not in another^19^. In light of the potential involvement of the reward system in AN^20–26^, the role of leptin as a strong modulator of this system deserves attention^5,14,16,27–30^ within the context of AN specific psychopathology.

We hypothesized that hyperactivity, but potentially also starvation-related emotional, cognitive, and somatic symptoms in patients with AN may be alleviated by off-label treatment with metreleptin^5^, a recombinant analog of human leptin. Metreleptin has been approved by the Food and Drug Administration and the European Medical Agency for the treatment of metabolic abnormalities in congenital or acquired generalized lipodystrophy^5,31,32^. Metreleptin has also been used to successfully treat a small number of patients with inborn leptin deficiency^33–35^, inducing a rapid reduction of hunger and substantial weight loss over time, as well as a normalization of metabolic and hormonal functions. Two studies, including a double-blind randomized controlled trial (RCT), revealed an improved reproductive function in females with hypothalamic amenorrhea^36,37^. Another RCT showed an increment of bone mineral content in strenuously exercising young women with hypoleptinemia^38^. In general, metreleptin is well tolerated. However, medium-term slight weight loss has been observed^36^, which in single patients can entail the cessation of treatment after several weeks^37,38^.

Based on the aforementioned considerations we for the first time treated three seriously ill patients with AN and pronounced hyperactivity with metreleptin. Such a case series, which according to German and Swiss regulatory requirements can only be conducted with a very limited number of patients outside of a clinical trial^39,40^, aimed to probe potential clinical effects of metreleptin treatment. Such efforts are clearly warranted in light of the overall limited effects of current off-label treatments for AN^41^. Thus, whereas drugs such as e.g. antidepressants^42^ and antipsychotics^43^ are widely prescribed for symptoms of AN, including hyperactivity^44,45^, the evidence-base for their effectiveness is meagre^2,42,43,46^.

## Patients and methods

### Setup and pre-treatment evaluation

**Fig. 1:**
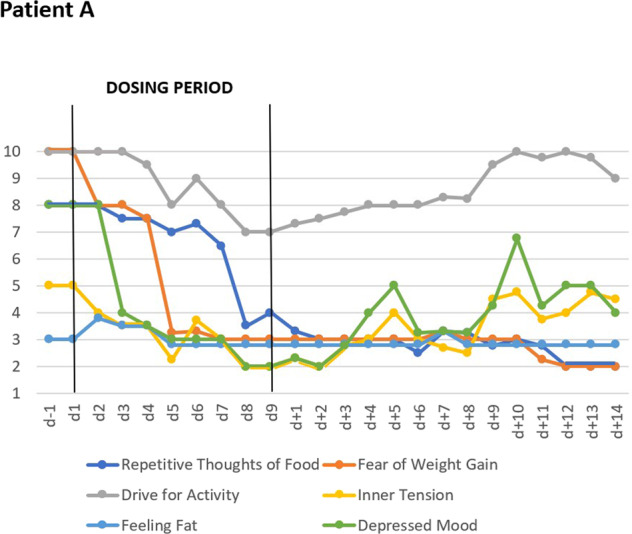
VAS for key cognitions and emotions: Effects of short-term metreleptin treatment in patient A including follow-up observations for 14-days, showing means of six key cognitions and emotions assessed thrice daily with visual analog scales (range 1–10).

**Fig. 2:**
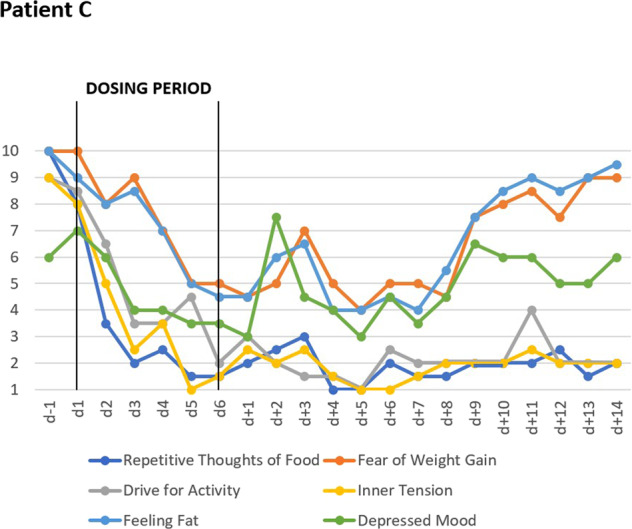
VAS for key cognitions and emotions: Effects of short-term metreleptin treatment in patient C including follow-up observations for 14-days, showing means of six key cognitions and emotions assessed twice daily with visual analog scales (range 1–10).

Two adult female inpatients (A, B) were treated at the Eating Disorders Unit, University Hospital Zurich, Switzerland, an adolescent female inpatient (C) at the Department of Child and Adolescent Psychiatry in Essen, Germany. All three patients suffered from intermittently life-threatening AN diagnosed according to DSM-5 (Table 1). This illness severity was also experienced by the patients themselves and represented the overarching inclusion criterion for the off-label treatment. All three patients subjectively complained about their drive for activity; the clinically apparent hyperactivity was rated as pronounced by the treatment teams (see Figs. 1 and 2, Supplemental Figs. 1 and 2, and clinical interview with patient A in Supplementary video; written informed consent was obtained).

**Table 1:** Descriptive case histories and clinical data of female patients A–C with anorexia nervosa treated with metreleptin.

			Patient		
			A	B	C
Type of AN (DSM-5)			Restricting	Restricting	Binge-eating/ purging
Family history			Maternal MDD and unspecified eating disorder	AN in maternal uncle	Parental obesity
Previous hospitalizations for AN		*N*	1	5	4
Age at		Years			
Referral			26	19	17
Onset of AN			15	13	14
Menarche			15	Primary amenorrhea	12
Maximum lifetime weight^a^			14	15	14
Minimum weight during AN			26	17	16
Weight		kg			
At referral			30.0	36.0	32.0
Maximum lifetime^b^			45.0	42.0	97.0
Minimum during AN			30.0	30.7	32.0
Height at referral		cm	162	164	166
BMI at		kg/m²			
Referral			11.4	13.4	11.6
Maximum lifetime^c^			17.1	15.6	35.2
Minimum during AN			11.4	11.4	11.6
*Metreleptin treatment*					
Dosing period	Days		9	14	6
Doses^d^	mg/day		4-6-7.5-10-10-0-10-0-10	2-2-3-3-4.5-6-6-8-0-10-0-11.3-0-11.3	6-9-9-9-9-9
Concurrent medication and daily doses			Aripiprazole 10 mg, fluoxetine 60 mg, diazepam 5 mg^e^, etilefrine hydrochloride 20 mg, multivitamin tablets with iron	Olanzapine 3.75 mg, sertraline 50 mg, phosphate 864 mg, multivitamin tablets	Olanzapine 2.5 mg, melperone 25 mg^f^
Selection criteria for metreleptin treatment			Severe AN; hyperactivity experienced as agonizing and compulsive; MDD	Severe AN; hyperactivity, MDD; palliative care considered in light of no weight gain after 12 weeks of current inpatient treatment episode including intermittent short-term medical stabilization in internal medicine unit	Severe AN; hyperactivity, MDD, recurrent episodes of life-threatening hyponatremia due to excessive drinking of water; stagnation of weight gain during inpatient treatment; premorbid obesity

*AN* anorexia nervosa, *BMI* body mass index, *MDD* major depressive disorder.

^a^Maximum body weight prior to metreleptin treatment

^b^Recalled body weight.

^c^BMI calculated using current height.

^d^Doses “0” indicates days during which treatment was discontinued.

^e^Diazepam discontinued by patient on day 4.

^f^Physician consented discontinuation of melperone and olanzapine during metreleptin treatment (days 3 and 4).

For patient B, the local therapeutic team had considered palliative treatment after consultation with a clinical ethicist because of treatment refractory AN. The initiation of dosing was postponed twice due to somatic instability. One day prior to dosing, she was transferred back to the Eating Disorders Unit after a 2-week treatment in the internal medicine ward, including nasogastric feeding, to achieve a stable somatic condition. In light of pre-relapse underweight in patient A, patient C was additionally selected for premorbid obesity. She was intermittently transferred to Essen for a total of twelve days from a hospital in the region. To allow for pre- and post-treatment assessment the dosing period lasted six days only.

Weight gain represented a central goal of the inpatient treatment regimens prior, during and after metreleptin treatment. Patients were treated as usual in the two interdisciplinary and multimodal eating disorder treatment programs and were requested to follow a defined daily meal plan including a specified energy intake to achieve weight gain. All patients received three main courses and three interim meals per day with between 2700 and 3000 kcal/day.

During the entire observation periods including (i) pre-dosing, (ii) dosing, and (iii) follow-up, body weights of patients were not measured daily; weights clearly vacillated throughout (Supplementary Table 1). We chose the closest weight measurements prior to and upon completion of dosing as body weights at T0 and T1 (see Table 2 and Supplementary Table 1); an exception due to excessive drinking to “reduce appetite” was made for patient C, for whom we used the realistic weight 2 days after end of dosing (d + 2). Prior to dosing, only two patients (A: +5.8 kg, C: +10.9 kg) had gained weight (Tables 1 and 2; Supplementary Table 1); patient C had, however, vacillated between 42 and 44 kg in the 4 weeks prior to dosing with a single peak at 45 kg due to water ingestion (see above).

**Table 2:** Safety, self- and clinician rated psychological data for patients A–C prior (T0) and at end of metreleptin treatment (T1) and serum leptin levels at T0 and during treatment.

		Patient					
		A		B		C	
		T0	T1	T0	T1	T0	T1
**Safety data**							
Body weight	kg	35.8	37.0	32.9	33.6	42.9	42.7
BMI	kg/m^2^	13.6	14.1	12.2	12.5	15.6	15.5
Pulse (range)		52–72	61–84	68–76	72–72	52–77	57–64
Systolic/diastolic blood pressure (range)	mmHg	85/55–90/70	85/55–95/60	90/65–95/60	80/60–90/60	75/60–113/58	100/60–105/70
Body temperature (range)	^o^C	36.2–37.1	36.1–37.3	35.2–36.0	35.6–36.8	36.5^a^	35.8^a^
Serum glucose (range)	mmol/l	4.6–5.1	5.1–6.5	3.8–4.3	4.8–5.1	4.8–6.2	4.1–6.6
Leucocytes	/nl	4.59	6.12	2.1	3.2	3.29	4.74
Lymphocytes	/nl	0.73	0.91	0.83	1.06	0.7	1.38
Erythrocytes	/nl	3.38	3.63	3.27	3.29	4.48	4.71
Thrombocytes	/nl	235	266	231	208	247	192
GOT	U/l	25	35	38	32	22	34
GPT	U/l	13	43	60	28	44	47
Amylase	U/l	70	68	44	53	54	69
Lipase	U/l	71	72	63	86	46	64
Electrocardio-gram		Sinus rhythm; NAD	Sinus rhythm; NAD	Sinus rhythm; NAD	Sinus rhythm; NAD	Sinus bradycardia; NAD	Sinus bradycardia; NAD
**Self-ratings**							
BDI-II		34	15	37	27	37	6
EDI-2	Percentile rank						
Total score		84	87	87	87	99	80
Drive for Thinness		75	75	85	90	99	75
Bulimia		1	99	1	1	99	45
Body Dissatisfaction		45	10	55	55	95	75
Ineffectiveness		99	85	95	95	99	80
Perfectionism		75	10	70	70	90	70
Interpersonal Distrust		95	55	80	85	60	50
Interoceptive Awareness		65	99	95	90	99	85
Maturity Fears		40	90	70	80	99	99
EDE-Q^b^							
Total score		3.11	2.48	3.62	3.53		
Restraint		2.4	2.4	3.2	3.4		
Eating Concern		1.2	0.6	2.4	2.4		
Weight Concern		3.6	1.8	3.8	3.2		
Shape Concern		5.25	5.13	4.88	5.13		
**Clinician ratings**							
HAMD-17		29	12	22	15	31	14
**Leptin serum levels**^**c**^							
T0	ng/ml	<0.5		<0.5		<0.1	

During treatment						3 time points:		
	ng/ml	72.9		4.4		6.7	55.9	80.8
Treatment day		7		13		2	4	5
Last dose	mg	10		11.3		6	9	9
Hours after sc. application^d^		7		22		22	2	4

NAD no abnormality detected, *BDI-II* Beck Depression Inventory-II, *BMI* body mass index, *EDI-2* Eating Disorder Inventory-2, *HAMD-17* Hamilton Depression Scale-17, *s.c* subcutaneous.

^a^Measured once daily only.

^b^Instructions were adapted to shortened observation time.

^c^Leptin assays for determination of total leptin serum concentrations: Leptin ELISA assay E07 (Mediagnost GmbH, Reutlingen, Germany; for patients A and B) and Leptin ELISA assay MD53001 (IBL International GmbH, Hamburg, Germany; patient C).

^d^For metreleptin a half-life of 3.8–4.7 h and a median *t*_max_ of 4 h (range 2–6 h) following s.c. administration was reported in patients with lipodystrophy^32^.

All contacted patients agreed to participate; a fourth patient who had been informed of the treatment, was unable to declare her unequivocal consent. Due to safety precautions, dosing periods were a priori limited to ten days with the possibility of an extension for an additional week in case of subtle treatment effects only.

All patients and parents of the 17-year old (C) provided written informed consent to off-label metreleptin treatment. Patient A received the information that the treatment aim was to reduce her severe hyperactivity. Based on these initial observations patients B and C were additionally informed about the possible antidepressant effect and the reduction of eating disorder specific cognitions. Patients (and parents) were instructed that non-adherence to requirements of the therapeutic team, including a specified daily total energy intake, could lead to discontinuation. The off-label treatment^5^ had been discussed and agreed upon by local and external physicians (G.M., J.H., S.T., N.B., R.v.K., M.W.); a medical ethicist (U.W.) provided ethical guidance. The off-label treatment was in accordance with the latest version of the Declaration of Helsinki^47,48^.

### Clinical assessment

All patients filled in a 10-item visual analog scale (VAS; scaled 1–10) for the assessment of key cognitions, emotions, and safety/physiological items twice or thrice daily (means presented in Figs. 1 and 2 and Supplementary Figs. 1 and 2 (patient B); no diurnal patterns for any item were observed); the ten items were ranked as follows: hunger, repetitive thoughts of food, fear of weight gain, drive for activity, inner tension, feeling full, nausea, feeling fat, depressed mood, tiredness. All patients were additionally assessed with the Hamilton Depression Scale-17^49^ (HAMD-17) by non-blinded clinical raters. Self-rating scales included Eating Disorders Inventory (EDI^50^; to allow comparisons with population-based norms we present percentile ranks^50,51^) and Beck Depression Inventory-II (BDI-II)^52^. Patients A and B also filled in the Eating Disorders Examination Questionnaire (EDE-Q)^53^; they were requested to base their answers on the last 7 days (instead of the last 28 days; instructions were adapted to the shortened observation time). Patients were clinically monitored during treatment (Table 2).

### Treatment

Metreleptin was applied subcutaneously (thigh) once daily at 9:30 am; dosage recommendations for patients with lipodystrophy served as guidance^32^. In patient A, the maximum dosage of 10 mg was reached at day four (Table 1). For patient B, who was the most acutely ill patient with the lowest body mass index (BMI) at baseline, the dosage was slowly titrated for safety concerns; she received the highest dosage of 11.3 mg on days twelve and fourteen. Based on the observed, uncomplicated courses of patients A and B, patient C was titrated to the maximal dose of nine mg at day two. Metreleptin was intermittently discontinued in patients A and B (Table 2).

## Results

**Fig. 3:**
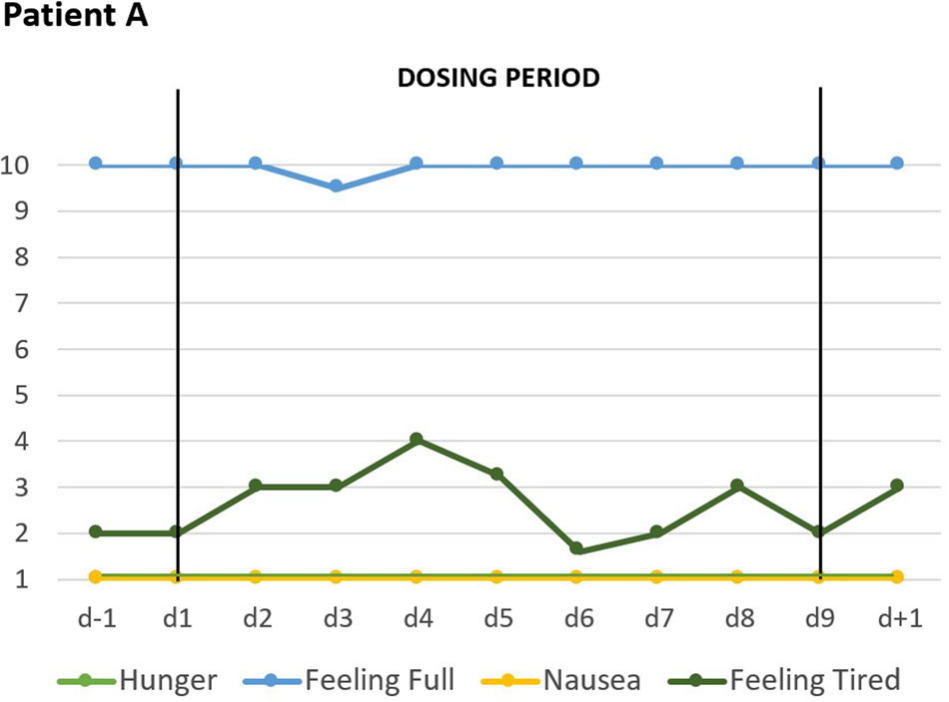
VAS for safety and physiology: Effects of short-term metreleptin treatment on four self-ranked safety/physiological parameters in patient A assessed thrice daily with visual analog scales (range 1–10).

**Fig. 4:**
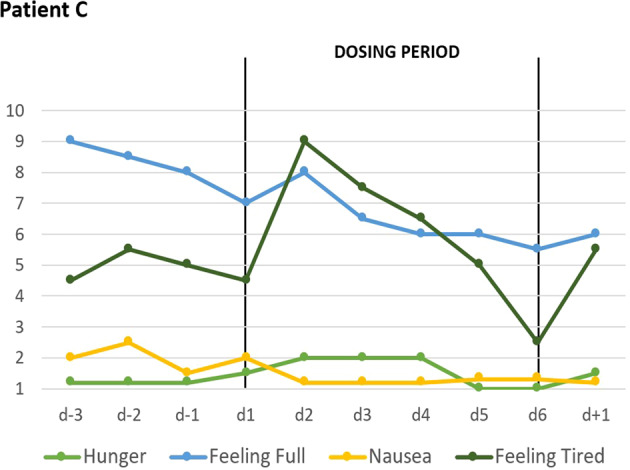
VAS for safety and physiology: Effects of short-term metreleptin treatment on four self-ranked safety/physiological parameters in patient C assessed twice daily with visual analog scales (range 1–10).

Two days after initiation of dosing, patients A and C began to rank most VAS items for key cognitions and emotions as less severe (Figs. 1 and 2). In the following days, the downward trend continued toward a plateau with low values. Whereas the overall effect was similar, quantitative differences were observed at the level of each item. Patient B reported no changes in the VAS (see Supplementary Fig. 1). Dosing did not affect self-rated (VAS; Figs. 3 and 4) safety/physiological items systematically in any patient.

During dosing periods, patients A and B gained 1200 and 700 grams, respectively; patient C lost 200 grams (Table 2); hunger was continuously rated as (almost) absent (Fig. 4). Fear of gaining weight, which was initially rated maximal by patient A, decreased during dosing (Fig. 1). Depressed mood, inner tension, and drive for activity increased in patient A during the 14 day long post-dosing observation period; fear of weight gain did not rebound. In patient C fear of weight gain and feeling fat rebounded (Fig. 2).

Self-rated depressive symptoms (BDI-II) decreased in all patients with patients A and especially C showing substantially lower values (Table 2). While the total EDI score improved only in patient C, the clinician-rated HAMD-17 revealed reductions in depressive symptoms in all patients. Patient A reported a clear improvement of her EDE-Q score; in patient B the improvement was marginal.

Circulating leptin levels (total-leptin), which were initially in the very low range characteristic for patients with acute AN^6^, reached high levels two to seven hours after metreleptin application, and were in the low normal range in the morning prior to the next dosing.

Increments were observed for leucocytes and lymphocytes in all patients. Pulse, blood pressure, body temperature, blood glucose, and other laboratory parameters revealed no systematic changes (Table 2).

Serious adverse events were not observed. In patient A, the upswing in mood was so pronounced, that dosing was discontinued on two days (Table 1). The patient herself reported a “happiness” equivalent to her lifetime maxima (see Supplementary video). She stopped taking diazepam because she no longer dreaded meals; she was surprised by the fact that she was able to phone lying relaxed in bed, for months she always did everything standing. Patient C experienced extended night sleeps and naps, which she experienced as healthy. Accordingly, treatment with melperone and olanzapine was terminated at days three and four, respectively.

Six months after treatment, patient A had achieved her lifetime maximal BMI of 20 kg/m^2^; her menses had resumed; she was well integrated in everyday life. She had intermittently developed panic attacks and a temporary episode of major depression, both of which had also occurred prior to referral. Patients B and C were discharged at days +33 and +24 due to failure to gain weight with BMIs of 12.4 kg/m^2^ and 15.9 kg/m^2^. Both are currently being treated on an outpatient basis.

### Clinical observations

During dosing, hyperactivity decreased only slightly in patient B, but substantially in patients A and C based on clinical observations of the treatment teams. For example, patient A was able to sit for 30 min for the purpose of an interview and intermittently lean back (see Supplementary video). Patient C, who would usually get up at 5 am prior to dosing to exercise, had to be wakened at 7.30 am after treatment for three days; she also took naps during the afternoon. Both patients reported a more “realistic” assessment of body shape and weight; they explained that metreleptin treatment allowed them to think outside the “cage” meaning that they were able to view themselves “realistically” without being constrained by their eating disorder. Both reported a substantial boost in their motivation to overcome AN, which patient A, but not patient C, was able to act upon. During dosing, patient C reported being able to discern her emotions more readily; prior to dosing she had been overwhelmed by them. According to her parents, she was substantially less hyperactive and agitated, her mood improved. She reported on dream contents unrelated to food. Despite low hunger ratings (Fig. 4), patient C reported an increase in “appetite” during treatment, which upset her in light of previous episodes of binge eating (see also increment in subscale Bulimia of EDI; Table 2). At the end of the dosing period she explained that this increment in appetite rendered her attitude toward metreleptin treatment more ambivalent despite the otherwise experienced substantial improvement.

All patients reported an improved ability to concentrate. They more readily initiated personal contacts, e.g. a renewed desire to contact friends and relatives (patient A), playing board games with other patients (B) and an intense, but fruitful, discussion with her parents (C).

Patient A reported less constipation (see Supplementary video). In patients A and C, the skin prior to subcutaneous application of metreleptin appeared wrinkly and dry. It rapidly normalized in turgor during dosing. Patient C observed healing and initial hair growth in bald spots due to excessive scratching.

## Discussion

Whereas the decreased drive for activity supported our primary hypothesis in our uncontrolled case series, the compelling and rapid changes of cognitive, emotional, and behavioral symptoms in patients A and C and to a markedly lower degree in patient B clearly warrant adequately powered double-blind and placebo-controlled RCTs. We had hypothesized beneficial effects on hyperactivity and starvation-related emotions and cognitions^5^, but were surprised by the rapid onset, the multiplicity of effects, the apparent effect sizes and the unexpected decrease in AN specific cognitions such as fear of weight gain and feeling fat. These effects seemingly allowed our patients an intermittent escape from the ‘golden cage’^54^ of their eating disorder. After cessation of metreleptin treatment most VAS items rebounded (Figs. 1 and 2).

In light of the lack of a consistent operational definition of hyperactivity in AN^5,55^, several different modes of assessment have been used with partially conflicting results. Because subjectively rated drive for activity may in part reflect muscle tension, inability to relax, inner restlessness, inner tension, and anxiety in addition to an elevated physical activity^55^, the use of pedometers or accelerometers does not necessarily capture the extent of the drive for activity. For the purpose of this case series, we used the VAS based subjective rating for drive for activity to obtain a subjective measurement. Obviously in future trials, both subjective and objective ratings should be used to capture the phenomena inherent to ‘hyperactivity´ (see ref. ^12^).

Patients A and B were able to gain weight during treatment; it cannot be excluded that the weight loss of 200 grams incurred during dosing in patient C may reflect an effect of metreleptin. However, both the short dosing periods and the weight vacillations prior and after treatment of patients B and C (Supplementary Table 1) preclude any definite conclusions as to an effect of metreleptin on body weight. Hunger was rated as absent or minimal throughout the total observation periods. It deserves notice that despite low hunger ratings (Fig. 4), patient C reported an increase in “appetite” during treatment, which clearly induced stress potentially in light of previous episodes of binge eating. We interpret her increased score on the EDI subscale Bulimia accordingly (Table 2). In contrast to the well-known anorexic function of leptin, we speculate that persistent hypoleptinemia may induce or contribute to absence of hunger; the therapeutically induced rapid resolution of hypoleptinema may however re-trigger appetite. If this speculation is correct, the treatment-induced appetite may render patients ambivalent as to the continuation of treatment.

Our somatic observations merit discussion. Increments in blood cell counts may reflect hematopoetic effects of leptin^56^. In the skin, leptin has been linked to cell differentiation, proliferation, migration, and survival with pronounced effects on angiogenesis, blood flow, and tissue perfusion, thus affecting skin aging, wound healing, and hair follicle morphogenesis^57^. Leptin also functions as an important modulator of gastrointestinal tract functions^58^.

In light of potential interactions, concurrent medications (Table 1) impede the interpretation of our findings as being due to metreleptin treatment only. However, it should be noted that two neuroleptic medications of patient C were stopped during treatment in light of the rapidly reduced hyperactivity and her emergent ability to rest. Patient A stopped pre-meal intake of diazepam, which she no longer deemed necessary.

The individualized metreleptin dosing schemes were based on safety considerations and ad hoc clinical observations. Importantly, for regulatory and ethical reasons, we were unable to consider matched, placebo treated controls. This major limitation entails that observed improvements may (partly) represent treatment expectation (placebo) effects. Indeed, all three patients were closely monitored and repeatedly interviewed entailing an unusual large amount of clinical attention. The subcutaneous mode of application may also have promoted expectation effects. However, for the following reasons we consider it highly unlikely that such effects in total explain our results: (i) In general, AN is one of the most difficult disorders in psychiatry to treat^2,59^; particularly achievement of weight gain is notoriously challenging in this disorder^4,60^. In clinical terms, we are unaware of any evidence for the occurrence of pronounced expectation effects in acutely ill patients. Thus, both verum and placebo effects have proven rather meager in AN^4^, indirectly reflected by the non-availability of any drug licensed for the treatment of this eating disorder. (ii) Patient A had only been informed (written informed consent) of a potential effect of metreleptin on hyperactivity; however, according to both her VAS rankings and clinical observations the rapid improvement of depression and the concomitant reduction of fear of weight gain was unexpected and pronounced. (iii) Some effects were observed in all three patients (improved mood, increased concentration, and social contacts). More subtle effects on concentration and social interaction (both not listed in the information sheets), were noticeable and reported by all three patients. (iv) Overall, the effects were similar in two patients (A and C) despite an age difference of ten years, their treatment in two different units and premorbid underweight versus obesity. (v) Interestingly, dosing did not affect the four safety/physiological items despite their listing on the same sheet as the other six items (for order of items see “Patients and methods”) indicating that patients did not merely rate all items as changed. (vi) Somatic effects consistent with current knowledge of the effects of leptin were observed, which cannot be explained by expectation effects. (vii) Overall the results were consistent with hypothesized effects of metreleptin treatment of patients with AN^5^. Results of a case series are to be judged as more reliable, if the underlying hypothesis is well founded and has been substantiated in e.g. rodent studies^61^ or related observations in humans^62^.

With respect to future systematic trials, important questions relate to treatment duration, minimal effective dose, medium-term safety and primary outcome criteria. Our patients might have profited from longer treatment durations. During dosing, patients A and C seemed more amenable to psychotherapy due to their elevated motivation and detachment from eating disordered cognitions. It remains to be seen to what extent the clinical changes enable longer-term behavioral changes and importantly weight gain. It will prove crucial to exclude a negative effect of metreleptin on weight gain in patients with AN. While weight gain will remain the focus of AN treatment, a shift toward improvement of quality of life as primary outcome in patients with longstanding or intractable AN has been proposed^63^. All of our patients had been ill for extended time periods entailing the need to rapidly adapt to the induced changes. Severity of starvation, pre-morbid body-weight (Table 1), and/or type of AN^1^ may account for short- and medium-term response variation. RCTs are required to assess whether (a) patients with shorter illness duration and less severe symptomatology may profit more, and (b) for how long “the cage needs to be unlocked” to allow patients—supported by standard treatment regimens—a more permanent ‘escape’ brought on by weight gain. The concomitant increase in fat mass should over time entail a sufficient endogenous leptin secretion to maintain the metreleptin induced behavioral, emotional, and cognitive improvements. Metreleptin treatment may initially help to overcome depression, reduce hyperactivity, and promote insight into the eating disorder pathology. The combined effect may increase the therapeutic motivation to more readily allow adjustment to the therapeutic regimen necessarily including a positive energy balance. As endogenous leptin secretion increases over a matter of weeks, metreleptin dosing could gradually be discontinued. Our results indirectly support weight rehabilitation as the mainstay of AN treatment^5^.

Our results suggest that leptin induced alterations at the post-receptor level result in clinically evident changes within a matter of 24–48 h. We also assume that as long as leptin levels stay above the critical level of ~2 µg/L (as threshold criterion for the initiation of the endocrine adaptation to starvation^64^, the restitution of a level well above this threshold is sufficient to maintain effects. Replication provided, AN may be recognized in part as a hormone deficiency syndrome. Hormones influence both cognitions and emotions with timing effects and critical periods^65^. The strong female preponderance of AN and the typical manifestation during the second decade of life may suggest such a sex-specific critical period. The seemingly different pathways, through which hypoleptinemia evokes emotional, cognitive, and behavioral derangements, need to be deciphered. In mice, leptin suppresses the rewarding effects of running via activation of signal transducer and activator of transcription-3 signaling in dopaminergic neurons of the ventral tegmental area without simultaneously affecting the anorectic actions of leptin and hedonic and compulsive feeding behavior^16^. The temporary evolution of specific cognitions, emotions, and behaviors during and after metreleptin treatment may help to elucidate if and how they are inter-related in patients. In a next step, it will be intriguing to investigate the central effects of metreleptin in terms of functional brain connectivity before and after treatment.

## Supplementary information

Supplementary Video of patient A part 1

Supplementa ary Video of patient A part 2

Supplementaryl Video of patient A part 3

Supplementary Table 1

supplementary figure legends

supplementary figure 1

supplementary figure 2
