# Supplementary material for: Short-term metreleptin treatment of patients with anorexia nervosa: rapid on-set of beneficial cognitive, emotional, and behavioral effects
Source: Transl Psychiatry. 2020 Aug 27;10:303. doi: 10.1038/s41398-020-00977-1 (PMC7453199; doi:10.1038/s41398-020-00977-1)
Supplement: Supplementary file 4 — Supplementary Table 1 [file 41398_2020_977_MOESM4_ESM.docx]

**Supplementary Table 1**

| **Patient A** | | |  | **Patient B** | | |  | **Patient C** | | |
| --- | --- | --- | --- | --- | --- | --- | --- | --- | --- | --- |
| **days** | **body weight (kg)** | **BMI (kg/m^2^)** |  | **days** | **body weight (kg)** | **BMI (kg/m^2^)** |  | **days** | **body weight (kg)** | **BMI (kg/m^2^)** |
| d-50 | 30.0 | 11.4 |  | d-115 | 36.0 | 13.4 |  | d-83 | 32.0 | 11.6 |
| d-48 | 30.3 | 11.5 |  | d-112 | 34.3 | 12.8 |  | d-51 | 39.70 | 14.4 |
| d-44 | 31.1 | 11.9 |  | d-105 | 34.1 | 12.7 |  | d-50 | 39.60 | 14.4 |
| d-42 | 31.8 | 12.1 |  | d-101 | 33.9 | 12.6 |  | d-49 | 41.00 | 14.9 |
| d-37 | 31.7 | 12.1 |  | d-98 | 33.9 | 12.6 |  | d-48 | 40.15 | 14.6 |
| d-34 | 32.5 | 12.4 |  | d-91 | 33.6 | 12.5 |  | d-47 | 40.60 | 14.7 |
| d-30 | 33.3 | 12.7 |  | d-87 | 33.9 | 12.6 |  | d-46 | 41.60 | 15.1 |
| d-27 | 33.4 | 12.7 |  | d-84 | 34.1 | 12.7 |  | d-45 | 42.20 | 15.3 |
| d-23 | 34.1 | 13.0 |  | d-80 | 32.7 | 12.2 |  | d-44 | 42.00 | 15.2 |
| d-20 | 34.2 | 13.0 |  | d-77 | 33.8 | 12.6 |  | d-43 | 40.80 | 14.8 |
| d-16 | 34.1 | 13.0 |  | d-73 | 33.5 | 12.5 |  | d-42 | 40.30 | 14.6 |
| d-13 | 34.7 | 13.2 |  | d-70 | 33.3 | 12.4 |  | d-41 | 40.40 | 14.7 |
| d-9 | 34.8 | 13.3 |  | d-66 | 33.1 | 12.3 |  | d-40 | 40.25 | 14.6 |
| d-6 | 35.5 | 13.5 |  | d-63 | 32.9 | 12.2 |  | d-39 | 40.50 | 14.7 |
| d-2 | 35.8 | 13.6 |  | d-59 | 33.1 | 12.3 |  | d-38 | 40.80 | 14.8 |
| d2 | 35.6 | 13.6 |  | d-56 | 32.8 | 12.2 |  | d-37 | 40.40 | 14.7 |
| d6 | 36.3 | 13.8 |  | d-52 | 33.3 | 12.4 |  | d-36 | 41.40 | 15.0 |
| d9 | 36.3 | 13.8 |  | d-49 | 33.1 | 12.3 |  | d-35 | 40.85 | 14.8 |
| d+3 | 37.0 | 14.1 |  | d-45 | 33 | 12.3 |  | d-34 | 40.70 | 14.8 |
| d+4 | 37.2 | 14.2 |  | d-42 | 32.9 | 12.2 |  | d-33 | 40.50 | 14.7 |
| d+7 | 38.2 | 14.6 |  | d-38 | 32.9 | 12.2 |  | d-32 | 41.00 | 14.9 |
| d+11 | 37.8 | 14.4 |  | d-35 | 32.3 | 12.0 |  | d-31 | 40.65 | 14.8 |
| d+14 | 38.4 | 14.6 |  | d-31 | 33 | 12.3 |  | d-30 | 40.70 | 14.8 |
| d+18 | 38.7 | 14.7 |  | d-24 | 32.1 | 11.9 |  | d-29 | 40.50 | 14.7 |
| d+21 | 39.6 | 15.1 |  | d-21 | 31.7 | 11.8 |  | d-28 | 40.40 | 14.7 |
| d+25 | 40.3 | 15.4 |  | d-1 | 34 | 12.6 |  | d-27 | 40.85 | 14.8 |
| d+32 | 42 | 16.0 |  | d1 | 32.9 | 12.2 |  | d-26 | 41.30 | 15.0 |
| d+35 | 42.3 | 16.1 |  | d5 | 33 | 12.3 |  | d-25 | 42.30 | 15.4 |
| d+39 | 42.8 | 16.3 |  | d7 | 33.2 | 12.3 |  | d-24 | 42.20 | 15.3 |
| d+42 | 43.6 | 16.6 |  | d8 | 33.1 | 12.3 |  | d-23 | 42.30 | 15.4 |
| d+46 | 44.7 | 17.0 |  | d10 | 33.5 | 12.5 |  | d-22 | 42.00 | 15.2 |
| d+49 | 44.9 | 17.1 |  | d12 | 33.8 | 12.6 |  | d-21 | 42.20 | 15.3 |
| d+53 | 45.7 | 17.4 |  | d14 | 33.4 | 12.4 |  | d-20 | 43.00 | 15.6 |
|  |  |  |  | d+1 | 33.6 | 12.5 |  | d-19 | 42.80 | 15.5 |
|  |  |  |  | d+3 | 33.5 | 12.5 |  | d-18 | 43.20 | 15.7 |
|  |  |  |  | d+5 | 33.6 | 12.5 |  | d-17 | 43.40 | 15.7 |
|  |  |  |  | d+12 | 34.2 | 12.7 |  | d-16 | 42.65 | 15.5 |
|  |  |  |  | d+15 | 34.1 | 12.7 |  | d-15 | 43.70 | 15.9 |
|  |  |  |  | d+19 | 33.7 | 12.5 |  | d-14 | 43.20 | 15.7 |
|  |  |  |  |  |  |  |  | d-13 | 42.90 | 15.6 |
|  |  |  |  |  |  |  |  | d-12 | 43.30 | 15.7 |
|  |  |  |  |  |  |  |  | d-11 | 42.80 | 15.5 |
|  |  |  |  |  |  |  |  | d-10 | 43.90 | 15.9 |
|  |  |  |  |  |  |  |  | d-9 | 43.40 | 15.7 |
|  |  |  |  |  |  |  |  | d-8 | 44.00 | 16.0 |
|  |  |  |  |  |  |  |  | d-7 | 45.10 | 16.4 |
|  |  |  |  |  |  |  |  | d-4 | 43.30 | 15.7 |
|  |  |  |  |  |  |  |  | d-3 | 41.30 | 15.0 |
|  |  |  |  |  |  |  |  | d-2 | 41.60 | 15.1 |
|  |  |  |  |  |  |  |  | d1 | 42.90 | 15.6 |
|  |  |  |  |  |  |  |  | d2 | 42.70 | 15.5 |
|  |  |  |  |  |  |  |  | d3 | 42.80 | 15.5 |
|  |  |  |  |  |  |  |  | d4 | 43.10 | 15.6 |
|  |  |  |  |  |  |  |  | d5 | 43.50 | 15.8 |
|  |  |  |  |  |  |  |  | d6 | 43.10 | 15.6 |
|  |  |  |  |  |  |  |  | d+1 | 44.50 | 16.2 |
|  |  |  |  |  |  |  |  | d+2 | 42.70 | 15.5 |
|  |  |  |  |  |  |  |  | d+3 | 43.00 | 15.6 |
|  |  |  |  |  |  |  |  | d+4 | 42.50 | 15.4 |
|  |  |  |  |  |  |  |  | d+5 | 42.70 | 15.5 |
|  |  |  |  |  |  |  |  | d+6 | 40.20 | 14.6 |
|  |  |  |  |  |  |  |  | d+7 | 43.70 | 15.9 |
|  |  |  |  |  |  |  |  | d+8 | 43.70 | 15.9 |
|  |  |  |  |  |  |  |  | d+9 | 43.10 | 15.6 |
|  |  |  |  |  |  |  |  | d+10 | 42.80 | 15.5 |
|  |  |  |  |  |  |  |  | d+11 | 42.80 | 15.5 |
|  |  |  |  |  |  |  |  | d+12 | 42.80 | 15.5 |
|  |  |  |  |  |  |  |  | d+13 | 43.20 | 15.7 |
|  |  |  |  |  |  |  |  | d+14 | 42.30 | 15.4 |
|  |  |  |  |  |  |  |  | d+15 | 43.30 | 15.7 |
|  |  |  |  |  |  |  |  | d+16 | 43.60 | 15.8 |
|  |  |  |  |  |  |  |  | d+17 | 43.65 | 15.8 |
|  |  |  |  |  |  |  |  | d+18 | 43.00 | 15.6 |
|  |  |  |  |  |  |  |  | d+19 | 42.90 | 15.6 |
|  |  |  |  |  |  |  |  | d+20 | 44.60 | 16.2 |
|  |  |  |  |  |  |  |  | d+21 | 43.60 | 15.8 |
|  |  |  |  |  |  |  |  | d+22 | 44.10 | 16.0 |
|  |  |  |  |  |  |  |  | d+23 | 43.90 | 15.9 |
|  |  |  |  |  |  |  |  | d+24 | 43.60 | 15.8 |

- Grey shaded areas indicate dosing periods.
- d-x indicates: days prior to dosing period. d+x indicates: days after dosing period / follow-up.
- BMI calculations based on height at referral: Patients: A = 1.62m. B=1.64m. C=1.66m
- Numbers marked in red indicate body weights at T0 and T1
