# Supplementary material for: Short-term metreleptin treatment of patients with anorexia nervosa: rapid on-set of beneficial cognitive, emotional, and behavioral effects
Source: Transl Psychiatry. 2020 Aug 27;10:303. doi: 10.1038/s41398-020-00977-1 (PMC7453199; doi:10.1038/s41398-020-00977-1)
Supplement: Supplementary file 5 — supplementary figure legends [file 41398_2020_977_MOESM5_ESM.docx]

**Legend to supplementary Fig. 1a)** Effects of short-term metreleptin treatment in patient B, showing means of six key cognitions and emotions assessed thrice daily with visual analogue scales (range 1-10).

**Legend to supplementary Fig. 1b)** Effects of short-term metreleptin treatment on four self-ranked safety/physiological parameters in patient B assessed thrice daily with visual analogue scales (range 1-10).

**Supplemental information to the submitted video**

Patient A agreed to have the anonymized video available online (written informed consent). She is the first patient with AN to have been treated with human recombinant leptin. The interviewer (JH) visited the Eating Disorder Unit of the University of Zürich (clinical head: GM) 24 hours after the last subcutaneous application of metreleptin. Upon entry to the ward the patient immediately introduced herself; the interviewer was struck by the atypical presentation of the patient. In particular, typical psychopathological features inherent to many patients with anorexia nervosa were lacking; this contrasted with her emaciated appearance. After a preliminary talk with the patient for approximately 15 minutes she agreed to a formal 30-minute-long interview in English (her native language is Italian); the interviewer had prior information which he used in the interview. The patient provides an insight into the effects of metreleptin treatment on motor hyperactivity, inner tension, mood and eating disorder specific cognitions. GM was in the same room and helped a few times to explain particular questions.

For technical reasons (size restrictions) the video had to be split in three parts.
