# Supplementary figures and images for: Short-term metreleptin treatment of patients with anorexia nervosa: rapid on-set of beneficial cognitive, emotional, and behavioral effects
Source: Transl Psychiatry. 2020 Aug 27;10:303. doi: 10.1038/s41398-020-00977-1 (PMC7453199; doi:10.1038/s41398-020-00977-1)

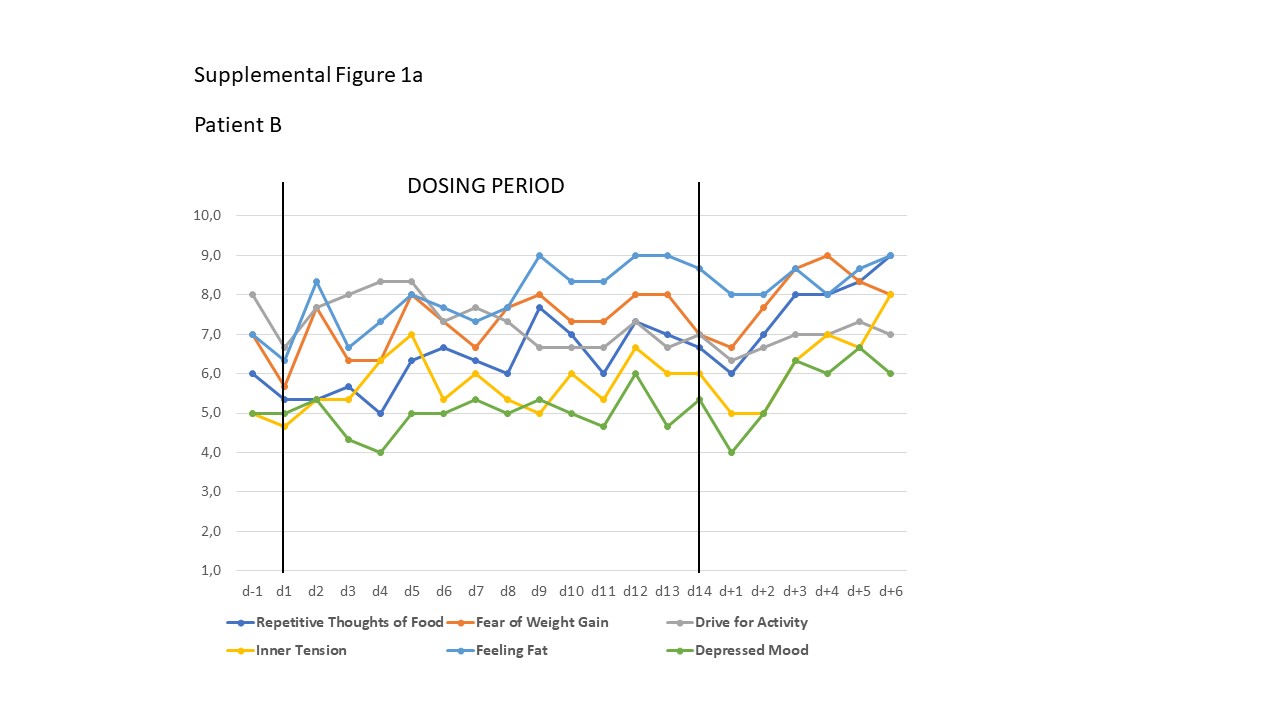

Supplement: Supplementary file 6 — supplementary figure 1 [file 41398_2020_977_MOESM6_ESM.jpg]

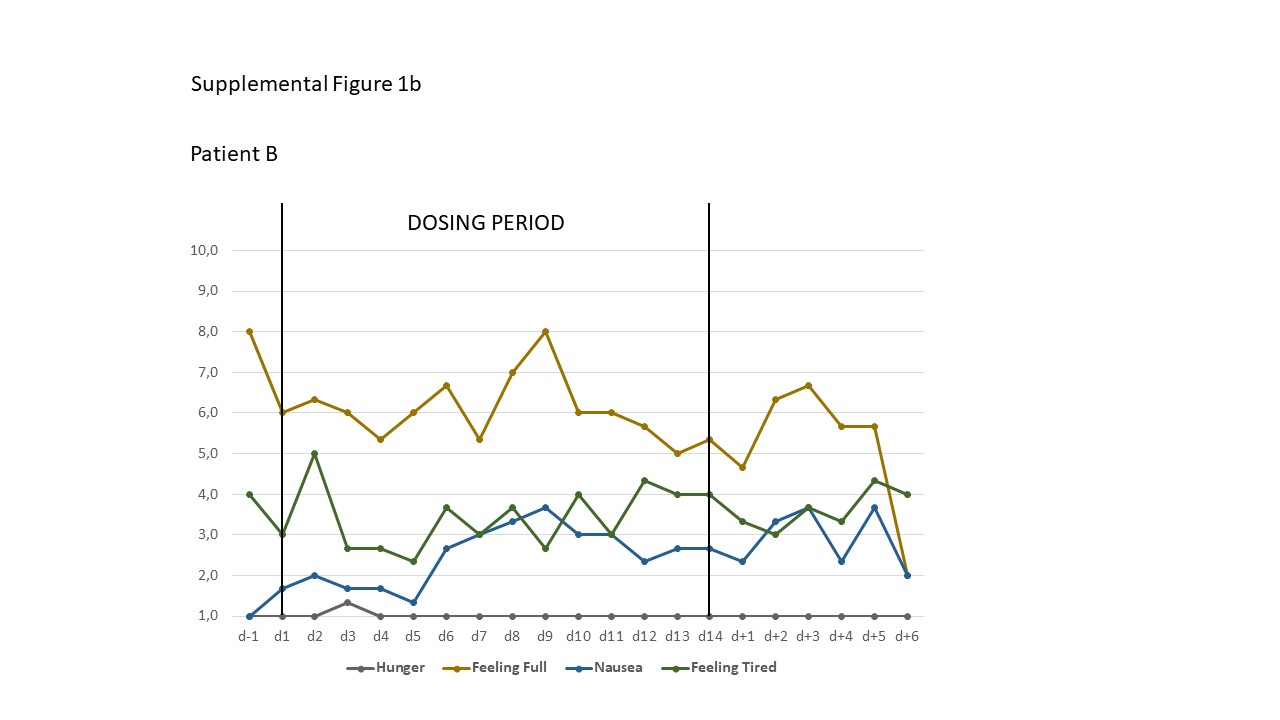

Supplement: Supplementary file 7 — supplementary figure 2 [file 41398_2020_977_MOESM7_ESM.jpg]
